# Supplementary material for: All-printed large-scale integrated circuits based on organic electrochemical transistors
Source: Nat Commun. 2019 Nov 7;10:5053. doi: 10.1038/s41467-019-13079-4 (PMC6838054; doi:10.1038/s41467-019-13079-4)
Supplement: Supplementary file 1 — Supplementary Information [file 41467_2019_13079_MOESM1_ESM.pdf]

Supplementary Information

All-Printed Large-Scale Integrated Circuits Based on Organic Electrochemical Transistors

Andersson Ersman et al.

Supplementary Figures

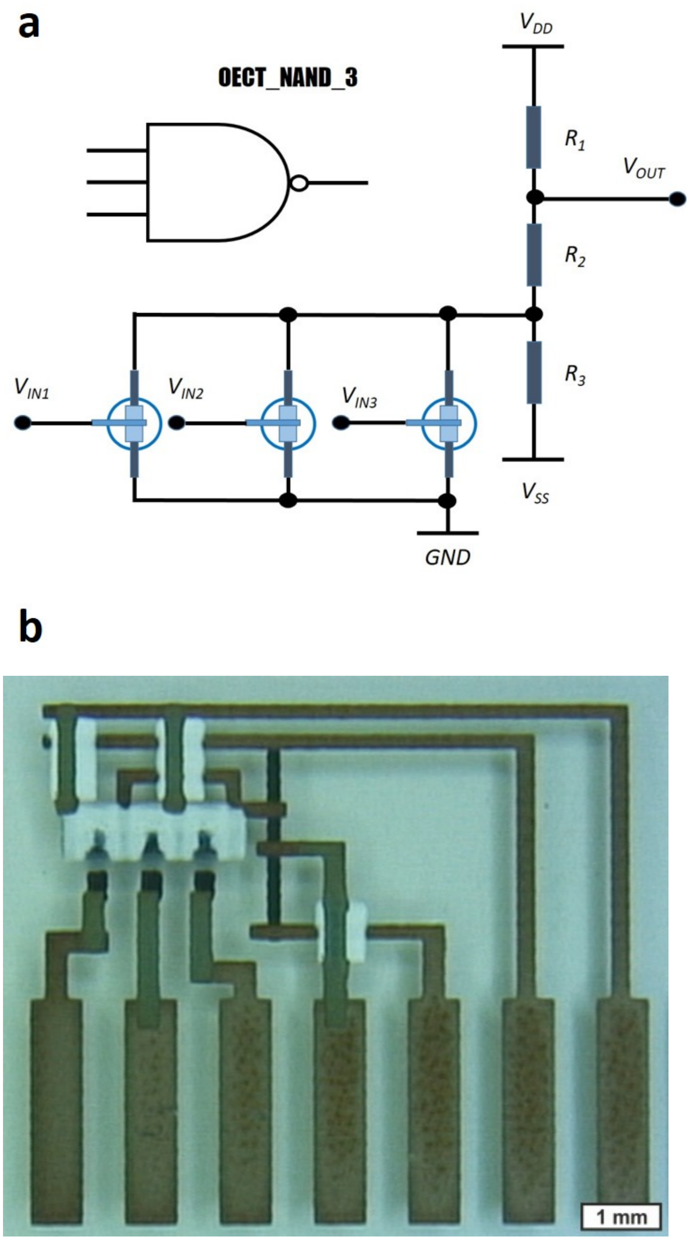

Supplementary Figure 1. Three-input NAND gate. **a** Schematics and **b** microscope photograph of a three-input NAND gate (scale bar: 1 mm).

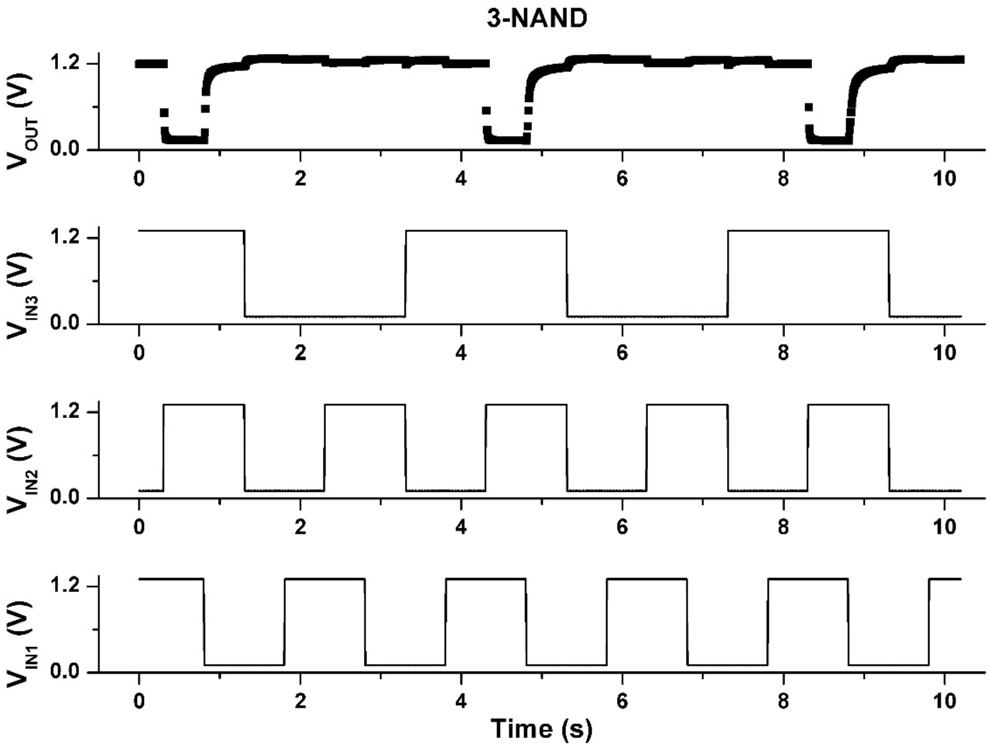

Supplementary Figure 2. Characteristics of a three-input NAND gate. The output HIGH voltage V_{OH} is >1.2 V with an input LOW voltage V_{IL} = 0.1 V, while the output LOW voltage V_{OL} is <0.13 V with an input HIGH voltage V_{IH} = 1.3 V.

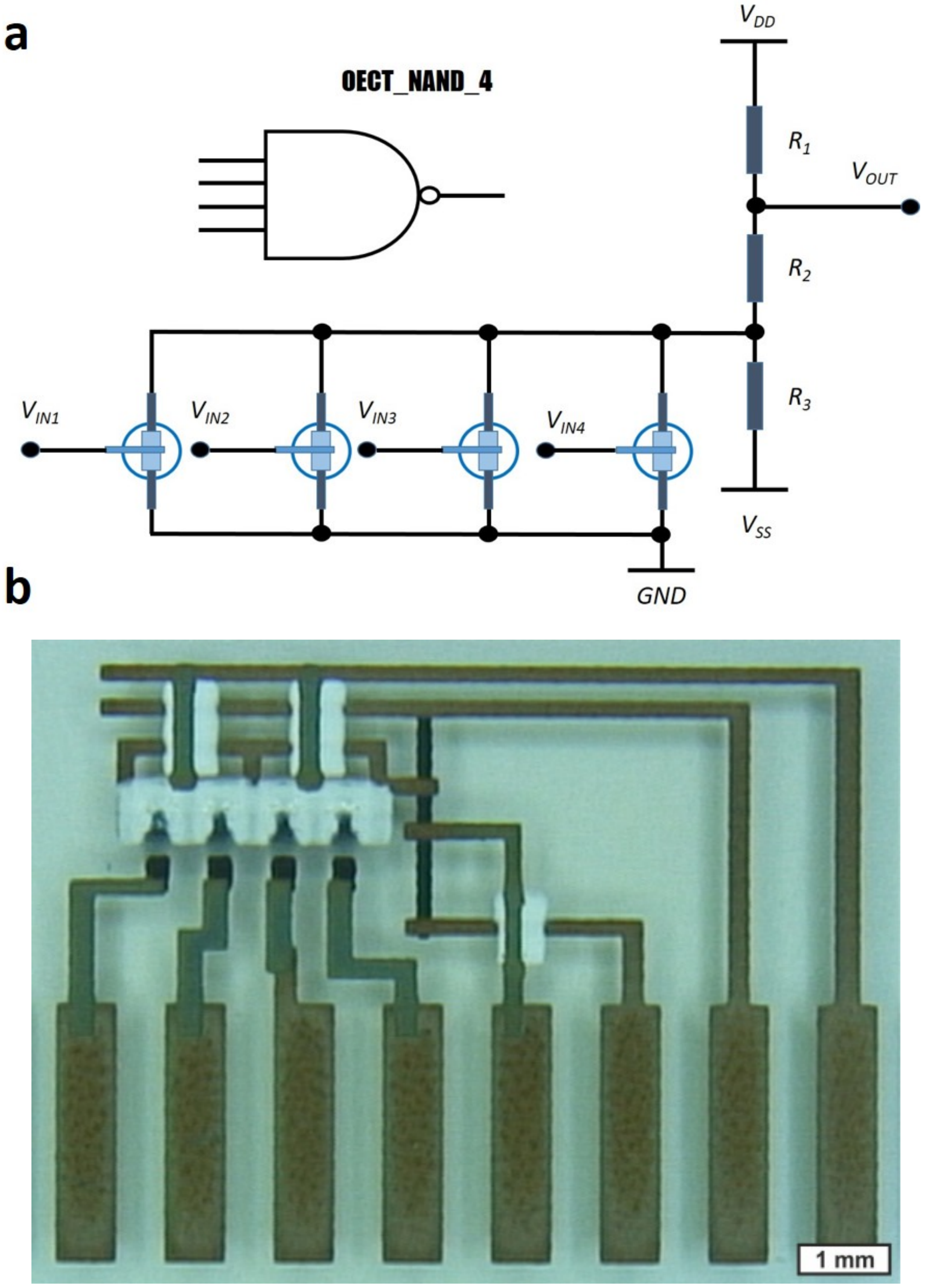

Supplementary Figure 3. Four-input NAND gate. **a** Schematics and **b** microscope photograph of a four-input NAND gate (scale bar: 1 mm).

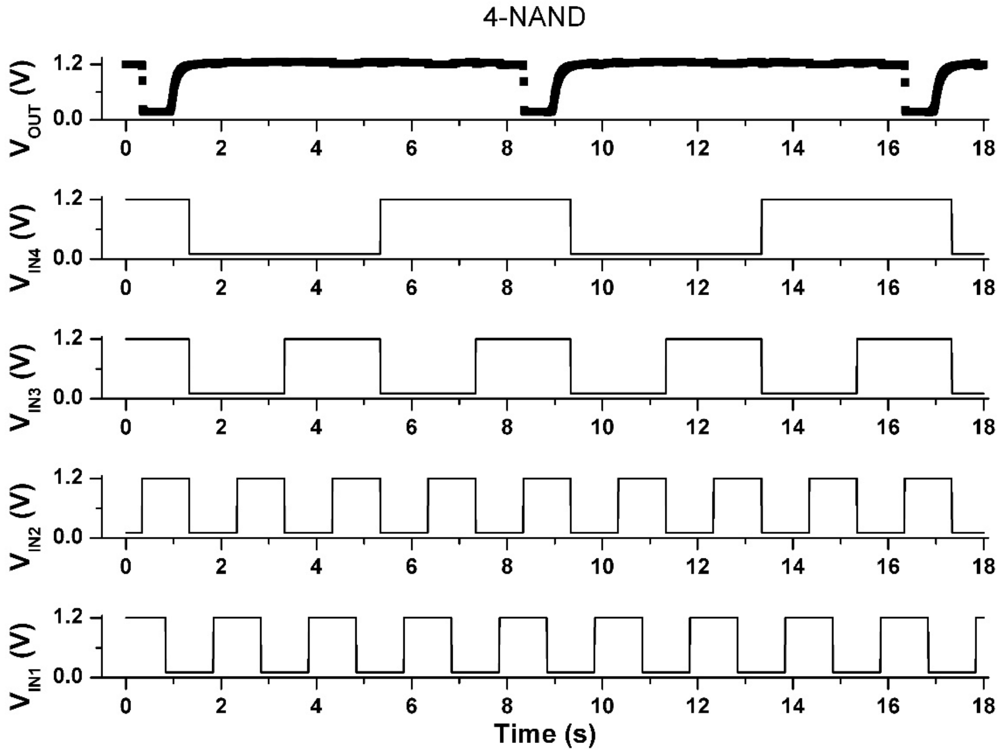

Supplementary Figure 4. Characteristics of a four-input NAND gate. The output HIGH voltage V_{OH} is >1.2 V with an input LOW voltage V_{IL} = 0.1 V, while the output LOW voltage V_{OL} is <0.17 V with an input HIGH voltage V_{IH} = 1.3 V.

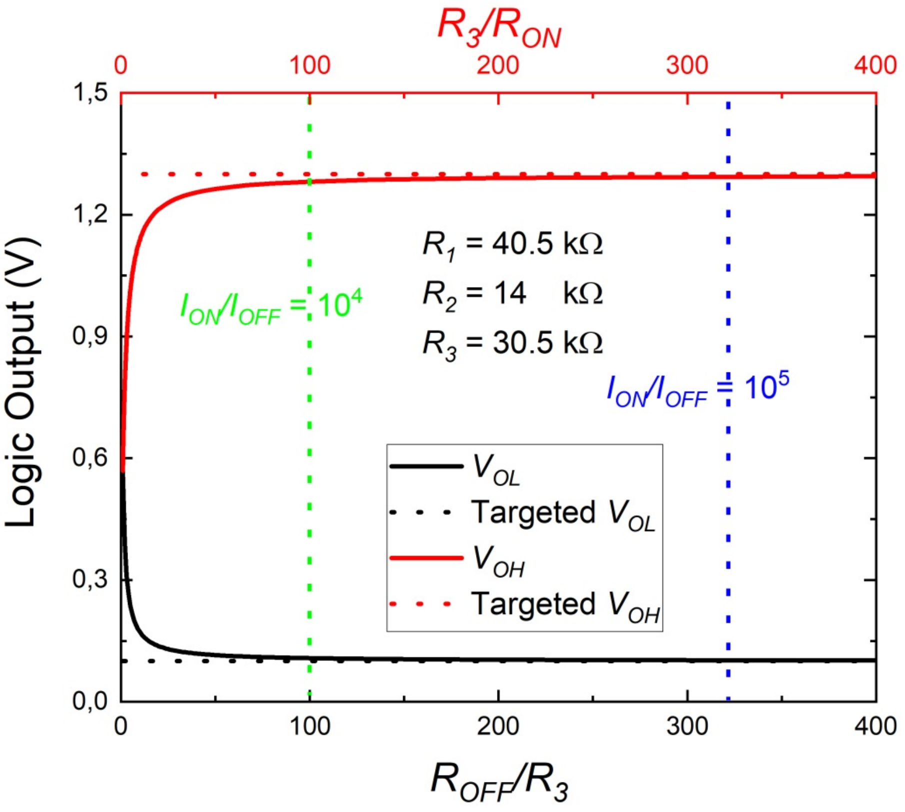

Supplementary Figure 5. Simulations describing how the output voltage of a logic circuit depends on the resistance of the OECT ON- and OFF-state in relation to R_3 in the resistor ladder. Even though an ON/OFF ratio exceeding 10^4 is desired, it is also evident that an ON/OFF ratio as low as ~400 would ensure reliable logic circuit operation, i.e. the resistance of the OFF-state (ON-state) should be at least a factor 20 higher (lower) than R_3.

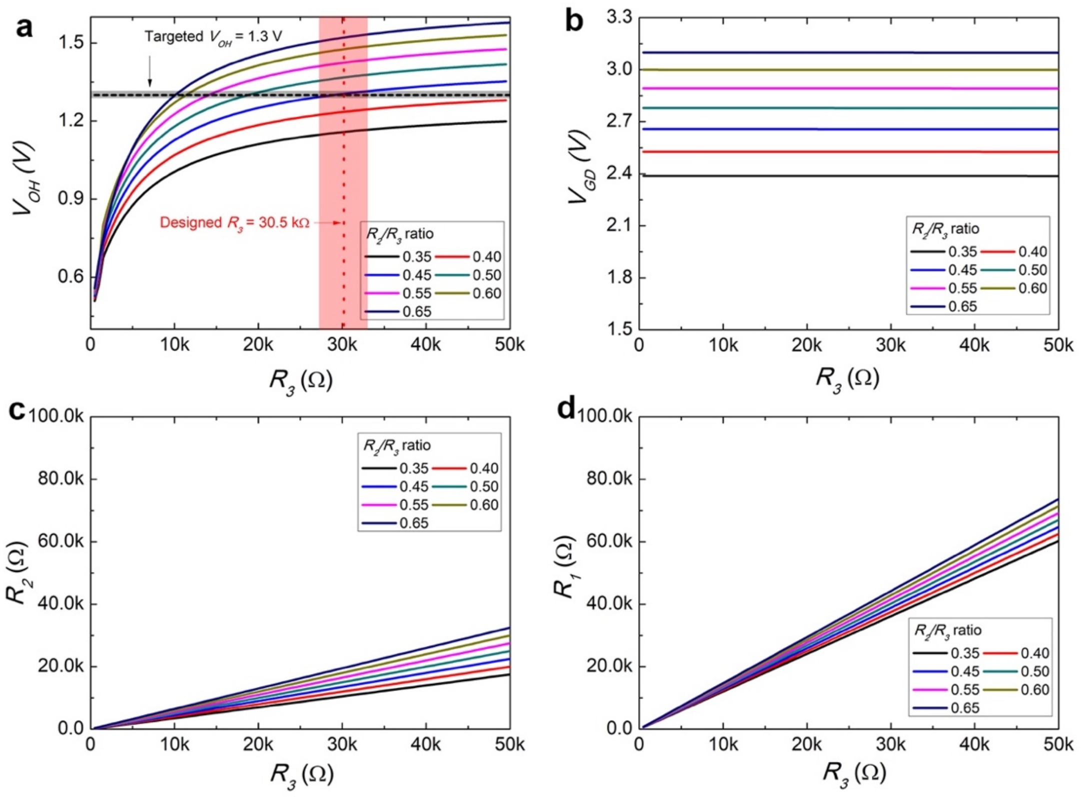

Supplementary Figure 6. Simulation results to obtain the target values of the resistors used in the resistor ladder; R_1 = 40.5 kΩ, R_2 = 14 kΩ, and R_3 = 30.5 kΩ. The simulations are based on a circuit power supply of +/-5 V and the obtained resistor values result in good switching performance by keeping the voltage strain at a relatively low level during circuit operation. **a** Simulation results showing the HIGH output voltage (V_{OH}) dependency on R_3. 10 % resistance variation of R_3 in the screen printing process (marked as red semi-transparent area), from the targeted 30.5 kΩ, results in only 1 % deviation from the targeted V_{OH} level at 1.3 V (marked as black semi-transparent area). **b** The simulated gate-drain voltage (V_{GD}), which reflects the voltage strain, as a function of R_3. The R_2 and R_1 dependencies on R_3 are shown in **c** and **d**, respectively.

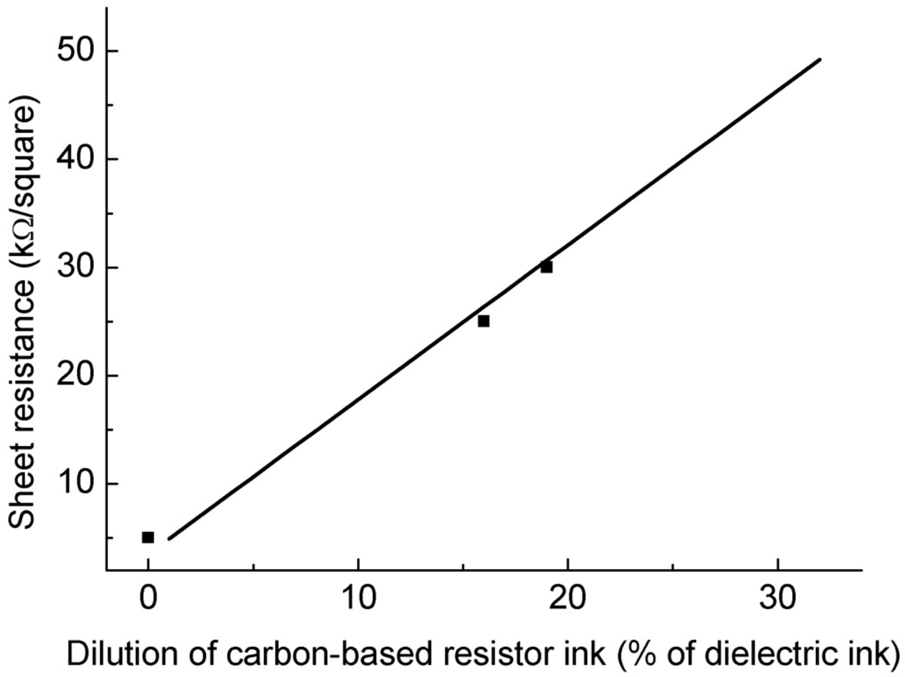

Supplementary Figure 7. Dilution of the carbon-based resistor ink, by adding an insulating dielectric ink, results in higher sheet resistance of the printed resistor structures. Here, almost 20 % of dielectric ink was added to the carbon-based resistor ink. This resulted in ~5 times higher sheet resistance, which in turn corresponds to a fivefold decrease in resistor footprint in the circuit designs.

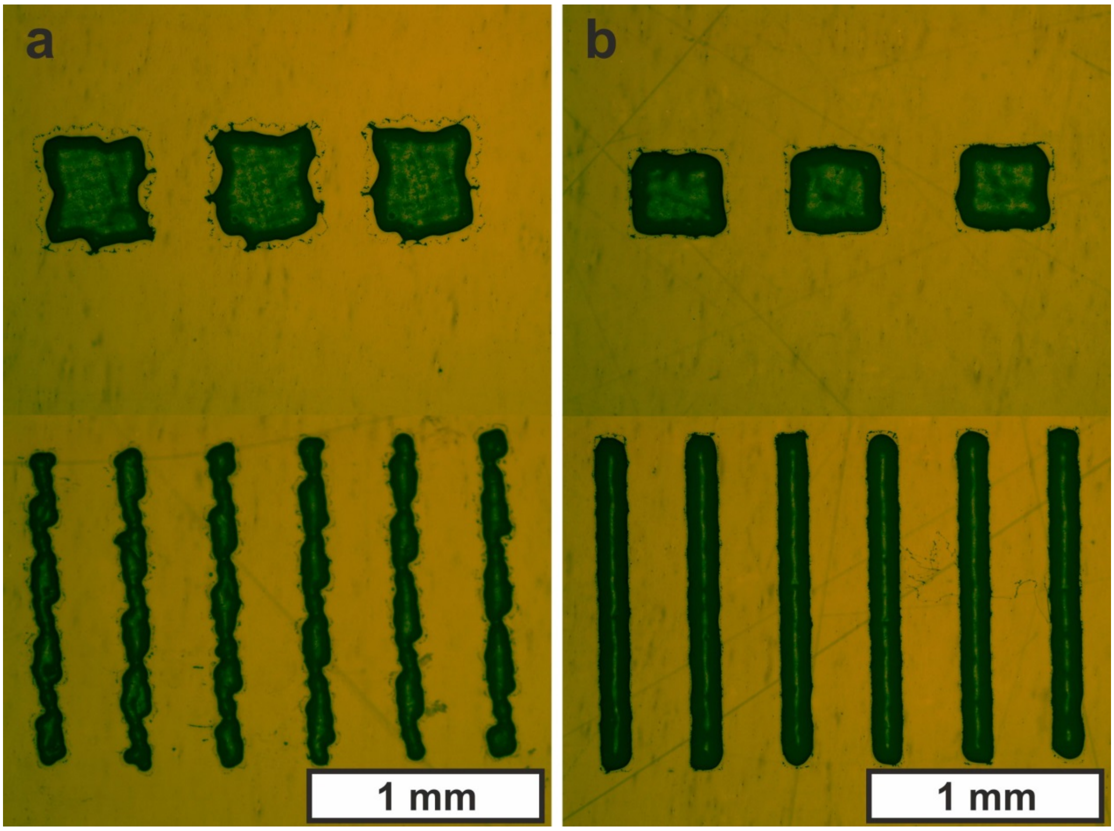

Supplementary Figure 8. Microscope images showing screen printed electrolyte patterns deposited by different screen printing tools (scale bars: 1 mm). **a** A mesh consisting of standard polyester threads has been used, which results in poorly defined electrolyte patterns. **b** Well-defined electrolyte patterns are obtained by using a mesh consisting of polyarylate-based threads, which in turn enables high manufacturing yield in the OECT screen printing process.

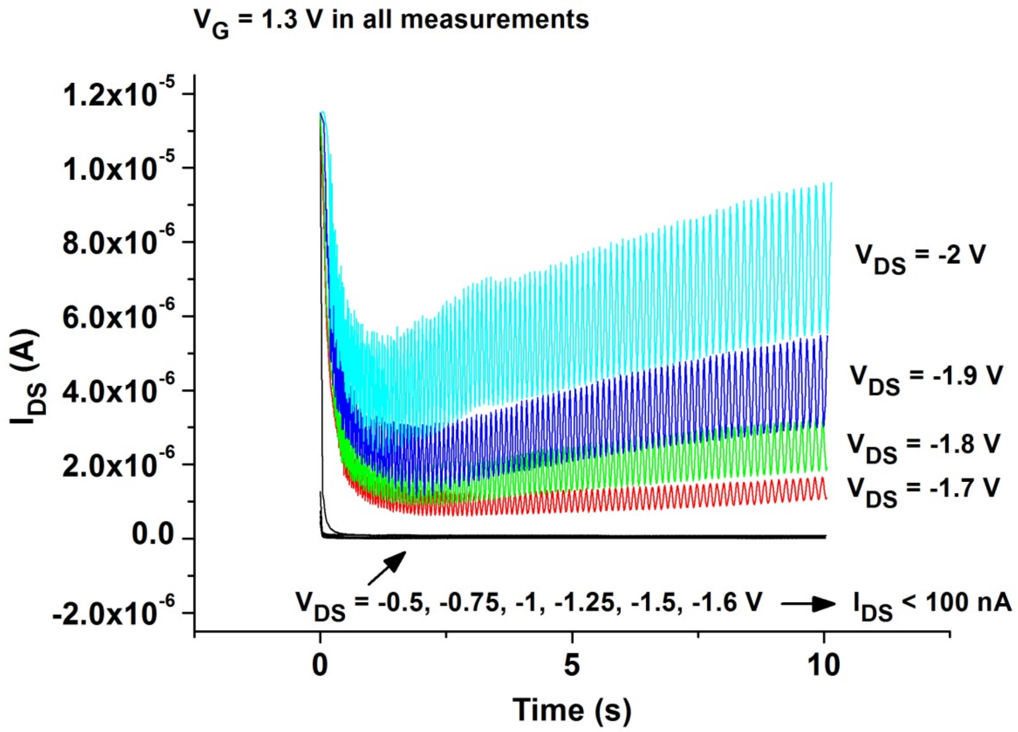

Supplementary Figure 9. Evolution of parasitic current levels for an OECT switched to its OFF-state, i.e. V_G is constantly set to 1.3 V in this measurement. For $V_{DS} \leq 1.6$ V, i.e. a maximum voltage strain of 2.9 V between the gate and drain electrodes, the current through the OECT channel remains lower than 100 nA, which is well below the required OFF-current level in circuit applications. For $V_{DS} \geq 1.7$ V, the OFF-current level is instead exceeding several μA, which inevitably will disable the circuit functionality. Hence, this adds an important criterion for the design of the resistor ladder. However, an exact criterion for the maximum current that is allowed through the channel in the OFF-state is difficult to define, since this also will depend on the circuit layout. For example, a four-input NAND gate, which is based on four OECTs connected in parallel, requires four times lower OFF-current as compared to an inverter based on only one OECT device. Note that the self-oscillations occurring at elevated voltages are outside the scope of this report [see Tu, D., Forchheimer, R. *Solid-State Electronics* **69**, 7-10, (2012) for a reference on self-oscillations in OECTs].

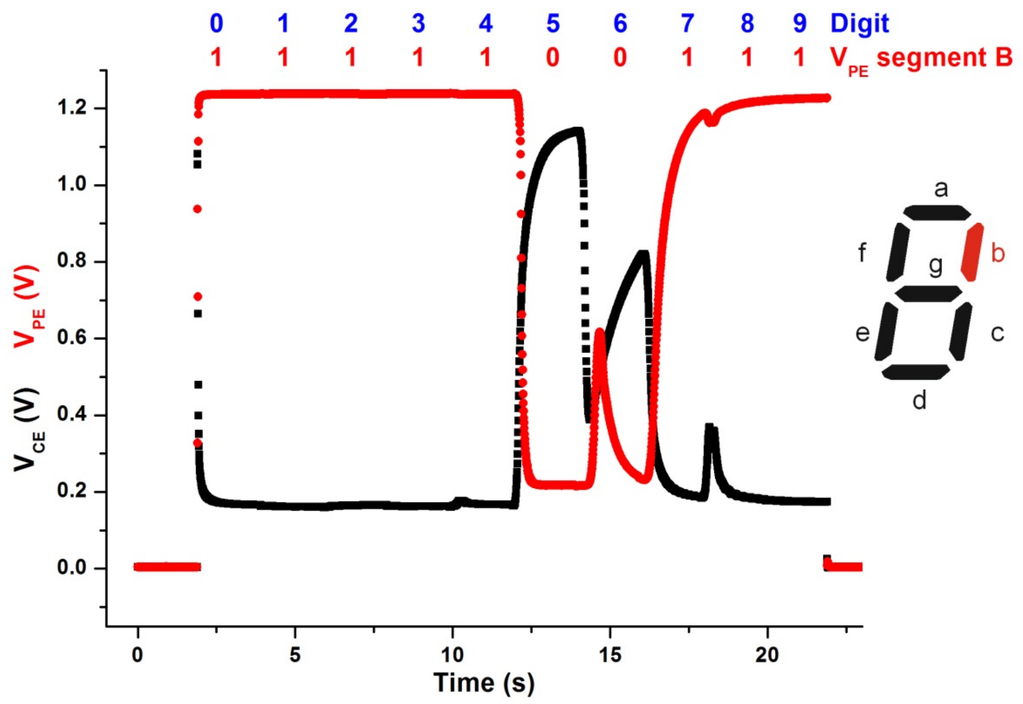

Supplementary Figure 10. Characterization of the 4-to-7 decoder, segment b. The voltage output sequence (V_{PE}, data shown in red is the logic level controlling the display pixel electrode) of segment b [1,1,1,1,1,0,0,1,1,1] when the input signal corresponds to the digits [0,1,2,3,4,5,6,7,8,9]. The data shown in black is the logic voltage level controlling the display counter electrode (V_{CE}).

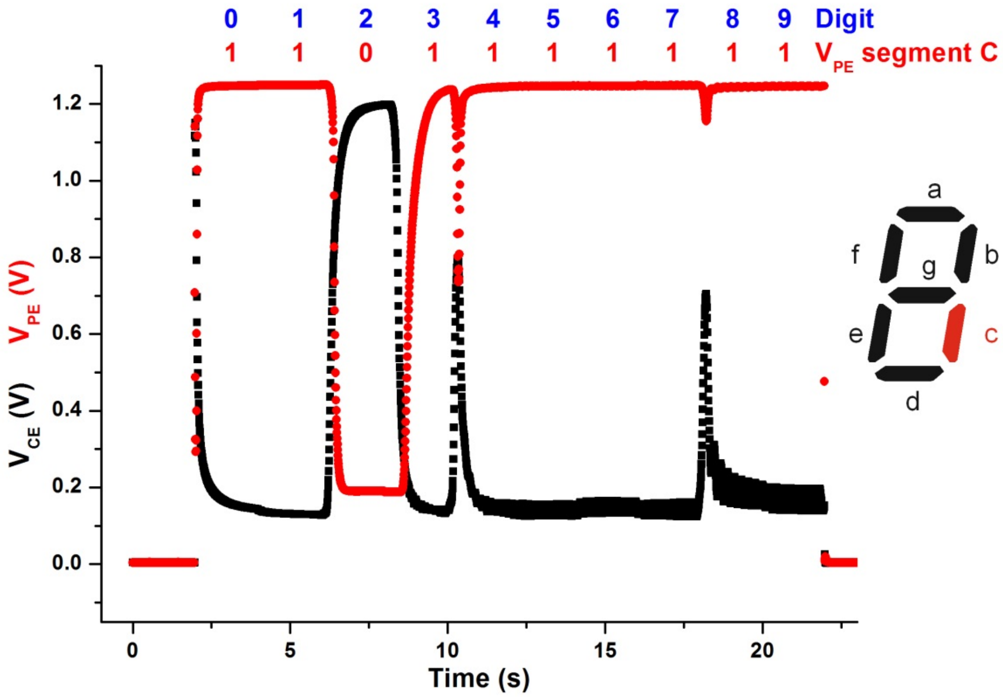

Supplementary Figure 11. Characterization of the 4-to-7 decoder, segment c. The voltage output sequence (V_{PE}, data shown in red is the logic level controlling the display pixel electrode) of segment c [1,1,0,1,1,1,1,1,1,1] when the input signal corresponds to the digits [0,1,2,3,4,5,6,7,8,9]. The data shown in black is the logic voltage level controlling the display counter electrode (V_{CE}).

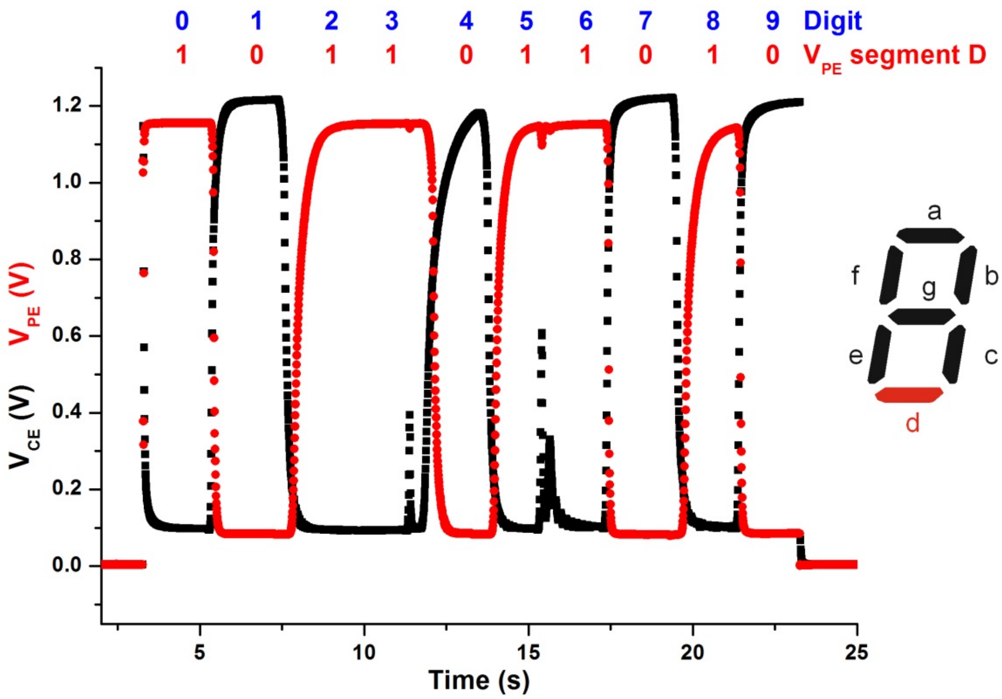

Supplementary Figure 12. Characterization of the 4-to-7 decoder, segment d. The voltage output sequence (V_{PE}, data shown in red is the logic level controlling the display pixel electrode) of segment d [1,0,1,1,0,1,1,0,1,0] when the input signal corresponds to the digits [0,1,2,3,4,5,6,7,8,9]. The data shown in black is the logic voltage level controlling the display counter electrode (V_{CE}).

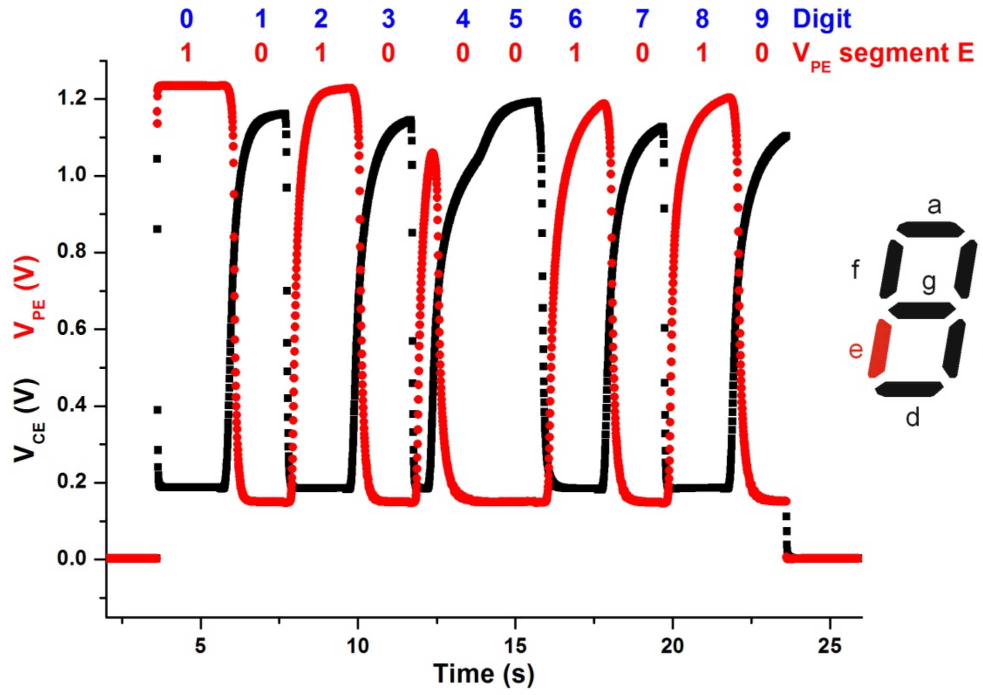

Supplementary Figure 13. Characterization of the 4-to-7 decoder, segment e. The voltage output sequence (V_{PE}, data shown in red is the logic level controlling the display pixel electrode) of segment e [1,0,1,0,0,0,1,0,1,0] when the input signal corresponds to the digits [0,1,2,3,4,5,6,7,8,9]. The data shown in black is the logic voltage level controlling the display counter electrode (V_{CE}).

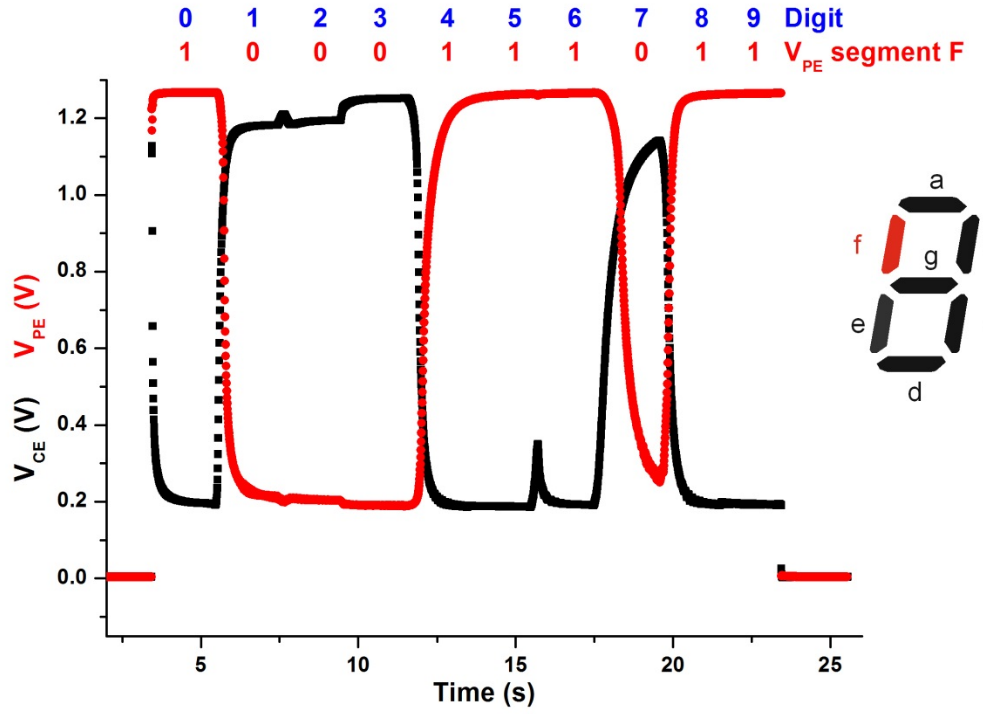

Supplementary Figure 14. Characterization of the 4-to-7 decoder, segment f. The voltage output sequence (V_{PE}, data shown in red is the logic level controlling the display pixel electrode) of segment f [1,0,0,0,1,1,1,0,1,1] when the input signal corresponds to the digits [0,1,2,3,4,5,6,7,8,9]. The data shown in black is the logic voltage level controlling the display counter electrode (V_{CE}).

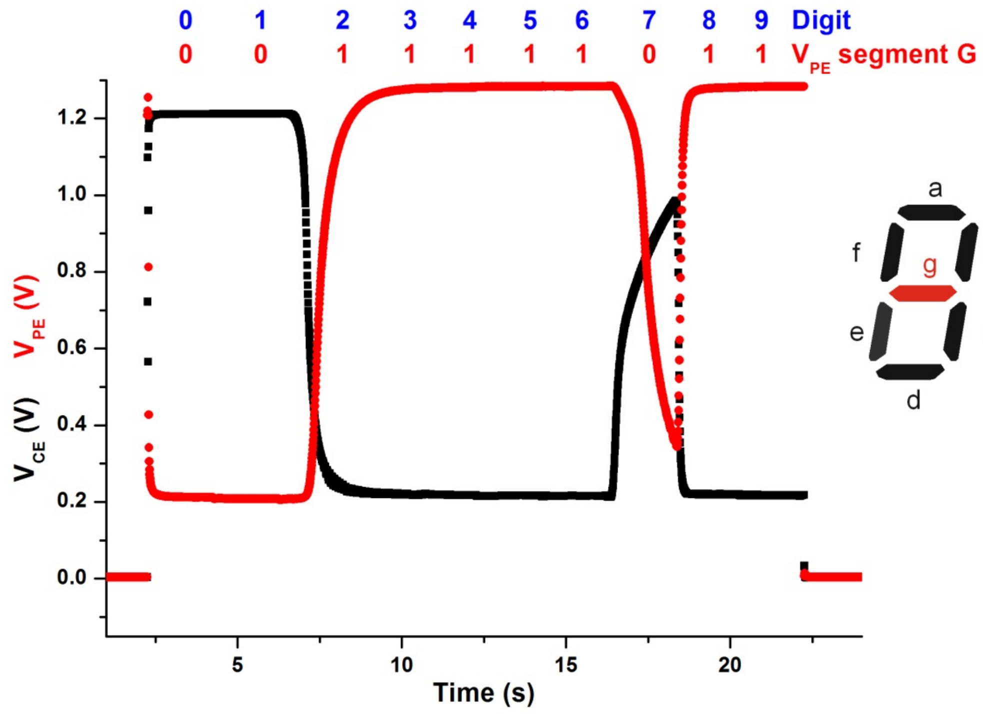

Supplementary Figure 15. Characterization of the 4-to-7 decoder, segment g. The voltage output sequence (V_{PE}, data shown in red is the logic level controlling the display pixel electrode) of segment g [0,0,1,1,1,1,1,0,1,1] when the input signal corresponds to the digits [0,1,2,3,4,5,6,7,8,9]. The data shown in black is the logic voltage level controlling the display counter electrode (V_{CE}).

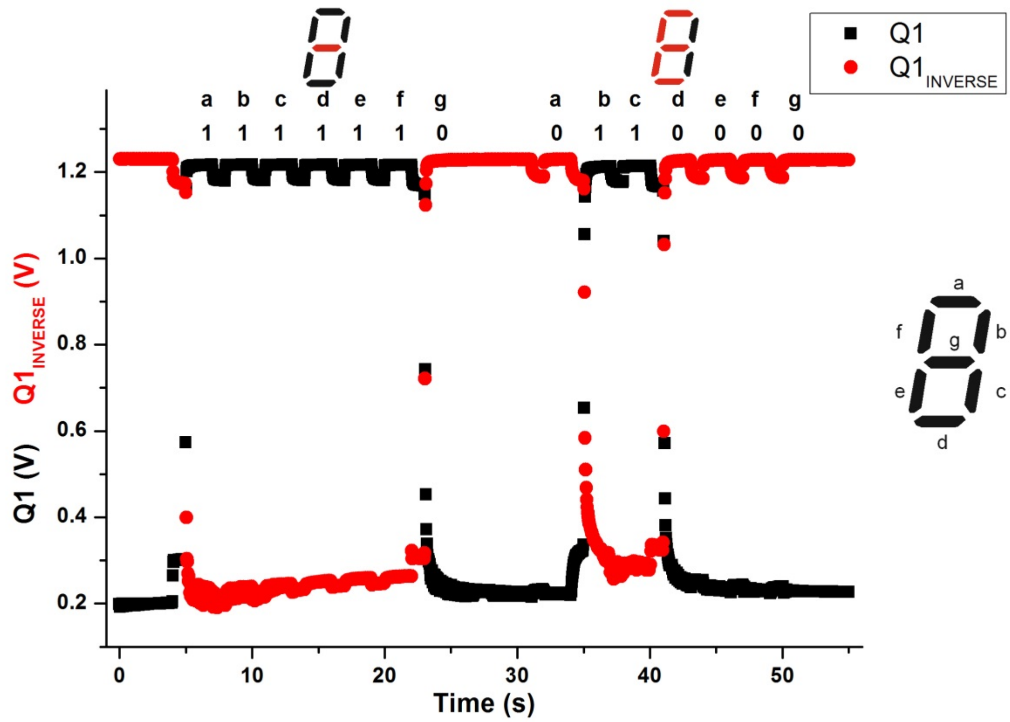

Supplementary Figure 16. Characterization of the 7-bit shift register, node g. The voltage output at node g (Q1 in black and its complementary $Q1_{INVERSE}$ in red), which is the first stage of the 7-bit shift register, when the input patterns [1,1,1,1,1,1,0] and [0,1,1,0,0,0,0] are shifted in seven clock cycles to display the digits [0, 1], respectively.

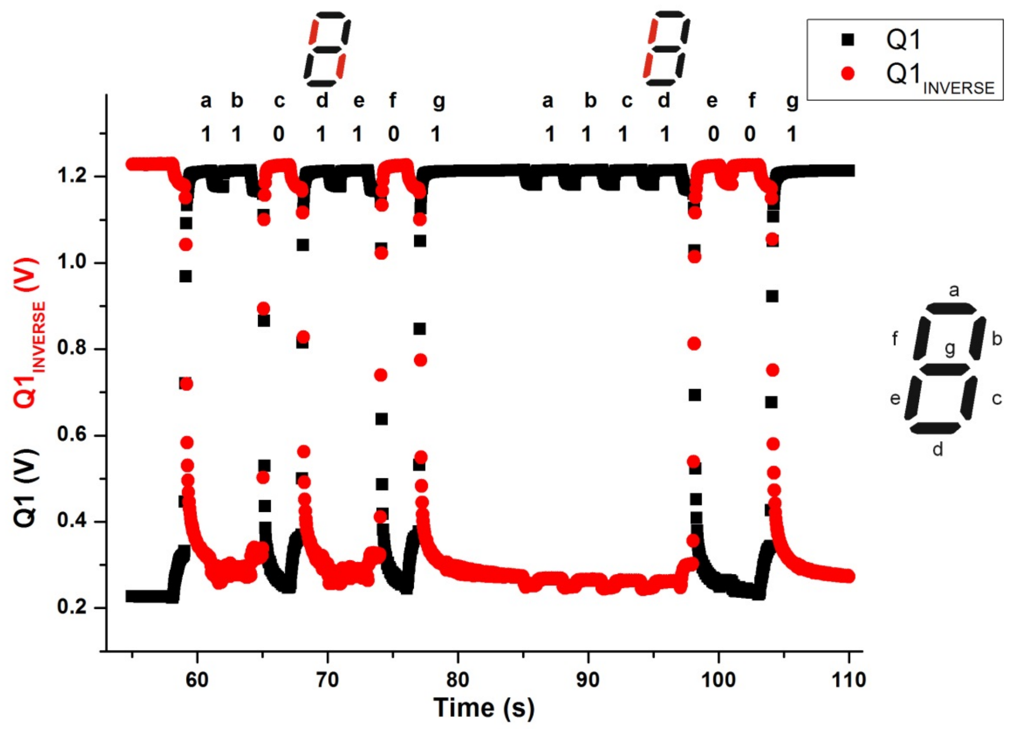

Supplementary Figure 17. Characterization of the 7-bit shift register, node g. The voltage output at node g (Q1 in black and its complementary $Q1_{INVERSE}$ in red), which is the first stage of the 7-bit shift register, when the input patterns [1,1,0,1,1,0,1] and [1,1,1,1,0,0,1] are shifted in seven clock cycles to display the digits [2, 3], respectively.

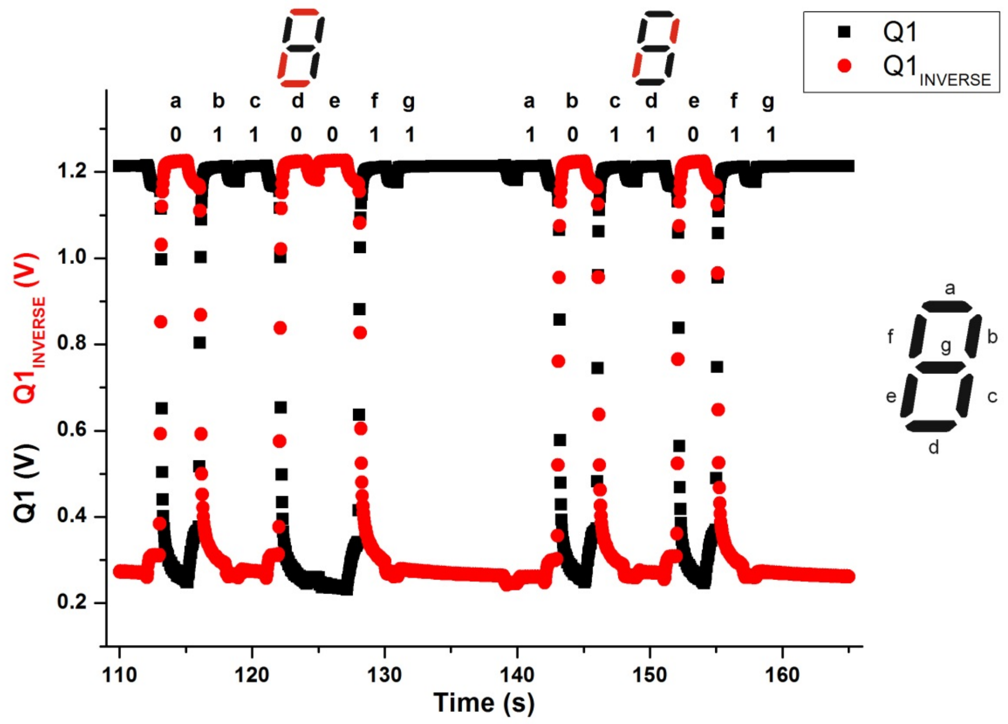

Supplementary Figure 18. Characterization of the 7-bit shift register, node g. The voltage output at node g (Q1 in black and its complementary $Q1_{INVERSE}$ in red), which is the first stage of the 7-bit shift register, when the input patterns [0,1,1,0,0,1,1] and [1,0,1,1,0,1,1] are shifted in seven clock cycles to display the digits [4, 5], respectively.

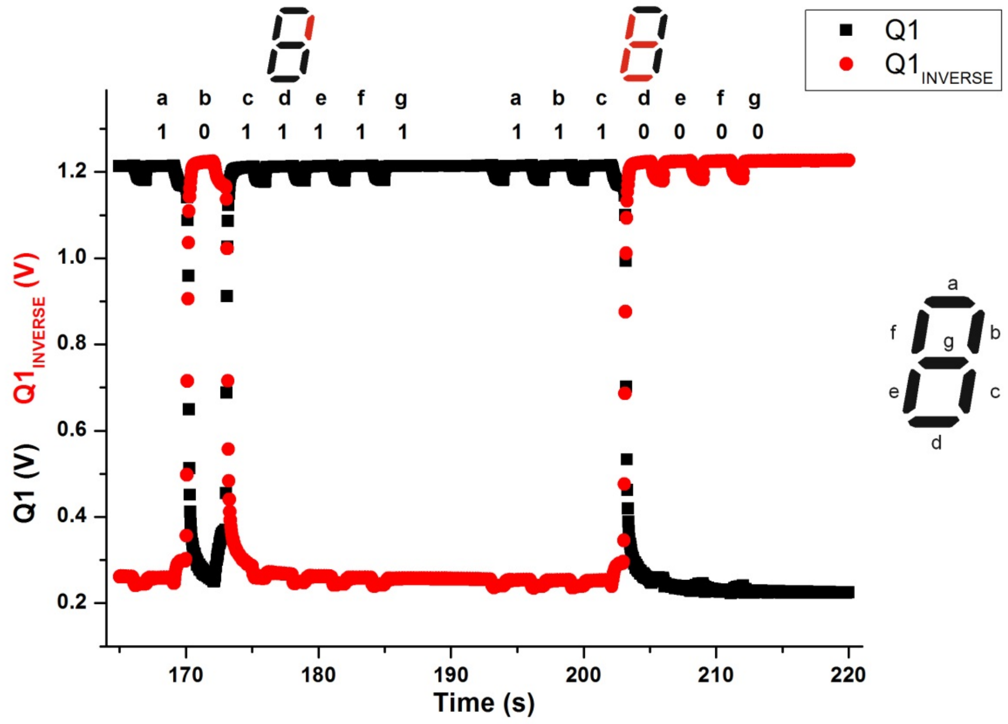

Supplementary Figure 19. Characterization of the 7-bit shift register, node g. The voltage output at node g (Q1 in black and its complementary $Q1_{INVERSE}$ in red), which is the first stage of the 7-bit shift register, when the input patterns [1,0,1,1,1,1,1] and [1,1,1,0,0,0,0] are shifted in seven clock cycles to display the digits [6, 7], respectively.

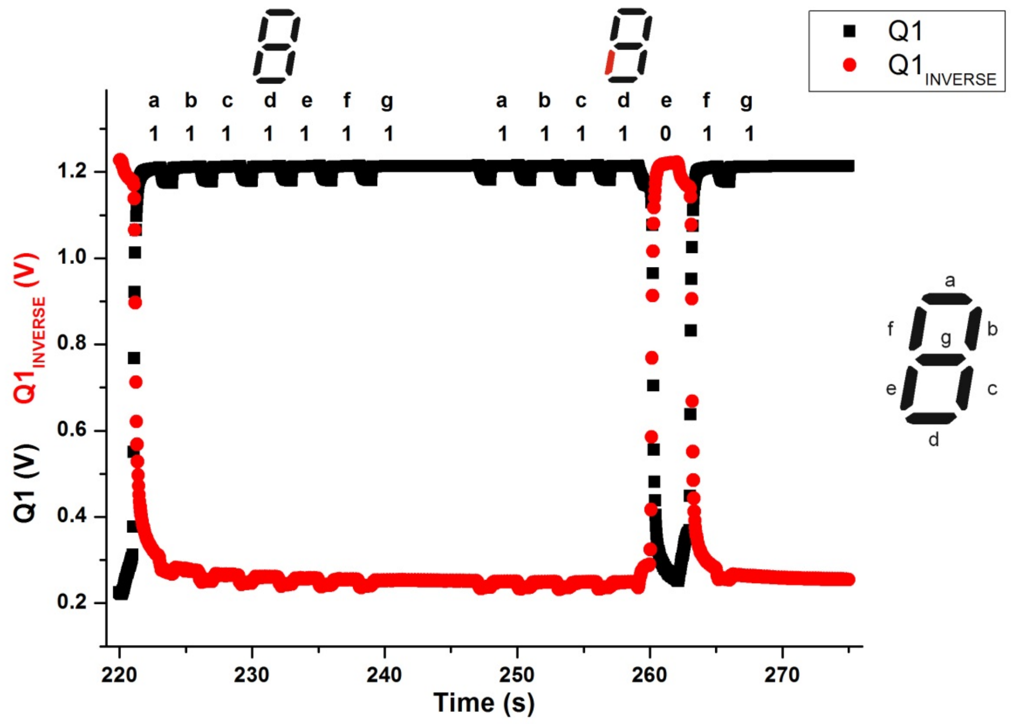

Supplementary Figure 20. Characterization of the 7-bit shift register, node g. The voltage output at node g (Q1 in black and its complementary $Q1_{INVERSE}$ in red), which is the first stage of the 7-bit shift register, when the input patterns [1,1,1,1,1,1,1] and [1,1,1,1,0,1,1] are shifted in seven clock cycles to display the digits [8, 9], respectively.
