# Supplementary material for: All-printed large-scale integrated circuits based on organic electrochemical transistors
Source: Nat Commun. 2019 Nov 7;10:5053. doi: 10.1038/s41467-019-13079-4 (PMC6838054; doi:10.1038/s41467-019-13079-4)
Supplement: Supplementary file 3 — Description of Additional Supplementary Files [file 41467_2019_13079_MOESM3_ESM.pdf]

Description of Additional Supplementary Files

File Name: Supplementary Movie 1

Description: The recorded video demonstrates that the 7-bit shift register is capable of delivering a logic ‘0’ in proper order from node g to node a. First, all seven electrochromic display segments are switched to their blue colored ON state by shifting in a logic ‘1’, this is achieved by using a digital HIGH voltage level as the serial input signal in combination with seven clock pulses. Then, a logic ‘0’ is shifted in, by using a digital LOW voltage level as the serial input signal in combination with seven clock pulses, which is evidenced by that the display segments are turned from their initial ON states (dark blue) to their OFF states, i.e. the ‘0’ bit is shifted one step for each clock pulse that is generated. It should be noted that the leftmost display segment shows weaker color contrast as compared to the other display segments, this is most probably caused by a pinhole (minor short-circuit) in the printed display segment. However, a slight color change can still be observed, which proves the functionality of the 7-bit shift register.

File Name: Supplementary Movie 2

Description: The recorded video presents that the all-printed 4-to-7 decoder drives an electrochromic 7-segment display to show all ten digits from 0 to 9. Note that the display segments in light grey present the digits while the dark blue represents display segments that are switched to their OFF states.
